# B7-H3 expression is associated with high PD-L1 expression in clear cell renal cell carcinoma and predicts poor prognosis

**DOI:** 10.1186/s13000-023-01320-0

**Published:** 2023-03-09

**Authors:** Jung Hee Lee, Yong Jun Kim, Hyun Woo Ryu, Seung Won Shin, Eun Ji Kim, So Hyun Shin, Joon Young Park, So Young Kim, Chung Su Hwang, Joo-Young Na, Dong Hoon Shin, Jee Yeon Kim, Hyun Jung Lee

**Affiliations:** 1 Department of Pathology, Pusan National University Yangsan Hospital, Pusan National University School of Medicine, Yangsan, Korea; 2 School of Medicine, Pusan National University, Yangsan, Korea; 3 The Research Institute for Convergence of Biomedical Science and Technology, Pusan National University Yangsan Hospital, Yangsan, Korea

**Keywords:** Clear cell renal cell carcinoma, Prognosis, B7-H3, PD-L1

## Abstract

**Background:**

Clear cell Renal cell carcinoma (ccRCC) is an immunogenic tumor. B7 family members, such as CTLA-4, PD-1, and PD-L1, are the main components of immune checkpoints that regulate various immune responses. Specifically, B7-H3 regulates T cell-mediated immune responses against cancer. This study aimed to analyze the association between B7-H3 and CTLA-4 expression and the prognostic factors of ccRCC to provide a basis for their potential use as predictive factors and in immunotherapy.

**Methods:**

Formalin-fixed paraffin-embedded specimens were obtained from 244 ccRCC patients, and B7-H3, CTLA-4, and PD-L1 expressions were evaluated using immunohistochemical staining.

**Results:**

B7-H3 and CTLA-4 were positive in 73 (29.9%) and 57 (23.4%) of the 244 patients, respectively. B7-H3 expression was significantly associated with PD-L1 expression (*P* <  0.0001); however, CTLA-4 expression was not (*P* = 0.842). Kaplan–Meier analysis showed that positive B7-H3 expression was associated with poor progression-free survival (PFS) (*P* <  0.0001), whereas CTLA-4 expression was not (*P* = 0.457). Multivariate analysis revealed that B7-H3 was correlated with poor PFS (*P* = 0.031), whereas CTLA-4 was not (*P* = 0.173).

**Conclusions:**

To the best of our knowledge, this study is the first to investigate B7-H3 and PD-L1 expression and survival in ccRCC. B7-H3 expression is an independent prognostic factor for ccRCC. Furthermore, multiple immune cell inhibitory targets, such as B7-H3 and PD-L1, can be used for therapeutic tumor regression in a clinical setting.

## Background

Clear cell renal cell carcinoma (ccRCC) accounts for 85% of renal cell carcinoma (RCC) tumors and is an immunogenic and proangiogenic cancer [1–3]. The dysfunction of immune cells, including tumor-associated macrophages, natural killer cells, antitumor cytotoxic T lymphocytes (CTLs), dendritic cells, and macrophages, and cytokine and chemokine action in tumor and stroma cells are essential to the pathogenesis of ccRCC [4].

ccRCC is an immunosensitive carcinoma that responds to immune checkpoint inhibitors [5–7] and is highly resistant to chemotherapy and radiotherapy [8, 9]. Therefore, immunotherapy is used as a major treatment for ccRCC.

B7 family T-cell costimulatory molecules play essential roles in modulating immune cell activation, function, and fate [10, 11]. B7 family members, such as CTLA-4, PD-1, and PD-L1, are the main components of the immune checkpoints, which positively or negatively regulate various immune responses. PD-L1 recognizes and attaches to the PD-1 of T cells, deactivating the T cells. Thus, PD-L1 on the surface of cancer cells prevents T cells from attacking them. PD-L1 is highly expressed in various carcinomas, and its expression is associated with a poor prognosis; thus, PD-L1 is used as a major immunotherapy target [12–14]. B7-H3 plays a role in regulating the T cell-mediated immune response against cancer. Recent evidence has shown that B7-H3 expression is positively associated with the density of FOXP3 + -regulated T cells, which infiltrate tumors [15].

CTLA-4 is expressed on the surface of T cells and binds to the B7 of antigen-presenting cells, inhibiting the activation signal of T cells. CTLA-4 is a negative regulator of T-cell immune function, as it stops potentially autoreactive T cells at the initial stage of naive T-cell activation, typically in the lymph nodes [16, 17].

Among the immune system participants introduced, PD-L1 is widely used as a biomarker. We aimed to discover a sensitive marker that can predict cancer prognosis and be applied to improve immunotherapy. The high correlations between the expression of B7-H3 and CTLA-4 and cancer cell activity can be used to evaluate their potential usefulness as prognostic predictors or immunotherapy targets.

Therefore, this study aimed to investigate the association between the expression of B7-H3 and CTLA-4 and the patterns and prognostic factors of ccRCC to provide a theoretical basis for the use of B7-H3 and CTLA-4 as predictive factors for ccRCC prognosis and as targets in ccRCC immunotherapy.

## Methods

### Patient selection

In total, 244 ccRCC patients treated with partial or total nephrectomy at Pusan National University Yangsan Hospital (Yangsan, Korea) between 2011 and 2017 were enrolled. Pathological diagnoses were made by genitourinary pathologists (H.J.L., J.H.L) and four medical students from the School of Medicine, Pusan National University. All patients were pathologically evaluated using the International Society of Urological Pathology/World Health Organization 2016 grading and pathological staging system.

### Immunohistochemical analysis

Immunohistochemistry was performed using formalin-fixed tissue samples obtained for the pathological diagnosis of ccRCC. High-density tissue microarrays (TMAs) were constructed using 244 ccRCC tissue samples. Each tumor was represented by 2 mm two cores for the highest and most common grade areas (total of 488 cores). A primary antibodies against B7-H3 (sc-376,769; Santa Cruz Biotechnology, Dallas, TX, USA) and CTLA-4 (ab237712; Abcam, Cambridge, MA, USA) were used. Sections were immunostained for B7-H3 with an anti-B7-H3 mouse monoclonal antibody (1:500) and for CTLA-4 with an anti-CTLA-4 rabbit monoclonal antibody (1:100).

Tumors were scored as positive, demonstrating membranous immune reactivity at low- and high-power magnifications. The B7-H3 immunostained samples were subdivided into two groups according to the macroscopic expression ratio (< 50% and ≥ 50%) on the TMA slides, regarded as negative and positive, respectively. The CTLA-4 immunostained samples were divided into two groups according to the macroscopic expression ratio (< 5% and ≥ 5%) on the TMA slides, regarded as negative and positive, respectively. PD-L1 expression was mainly confined to the tumor cell membrane. PD-L1 tumor positivity was defined as ≥5% tumor cell membrane staining [12].

### Statistical analysis

The relationship between clinical and pathological features was evaluated using the χ^2^ test. The PFS rate was estimated using the Kaplan–Meier method for univariate analysis and the Cox proportional hazards regression model for multivariate analysis. PFS was defined as the duration between the date of surgery and the date of recurrence or metastasis. Statistical analyses were performed using Statistical Package for the Social Sciences version 19.0 (SPSS, Inc., Chicago, IL, USA).

## Results

### Patient characteristics

The patient cohort consisted of 183 male and 61 female study participants, with a median age of 61 years (range, 27–88 years). None of the patients had a history of malignancy. The follow-up was 1–138 months, and the median PFS was 77 months (Table 1).

**Table 1:** Patient characteristics

Characteristic	Number of patients (%) Entire cohort (*N* = 244)	
Sex		
Male	183	(75.0)
Female	61	(25.0)
Age		
≤ 50 years	47	(19.3)
> 50 years	197	(80.7)
Tumor stage		
I	168	(68.9)
II	22	(9.0)
III	52	(21.3)
IV	2	(0.8)
ISUP grade		
1	5	(2.1)
2	129	(52.9)
3	95	(38.9)
4	15	(6.1)

### B7-H3 and CTLA-4 expression in ccRCC

**Fig. 1:**
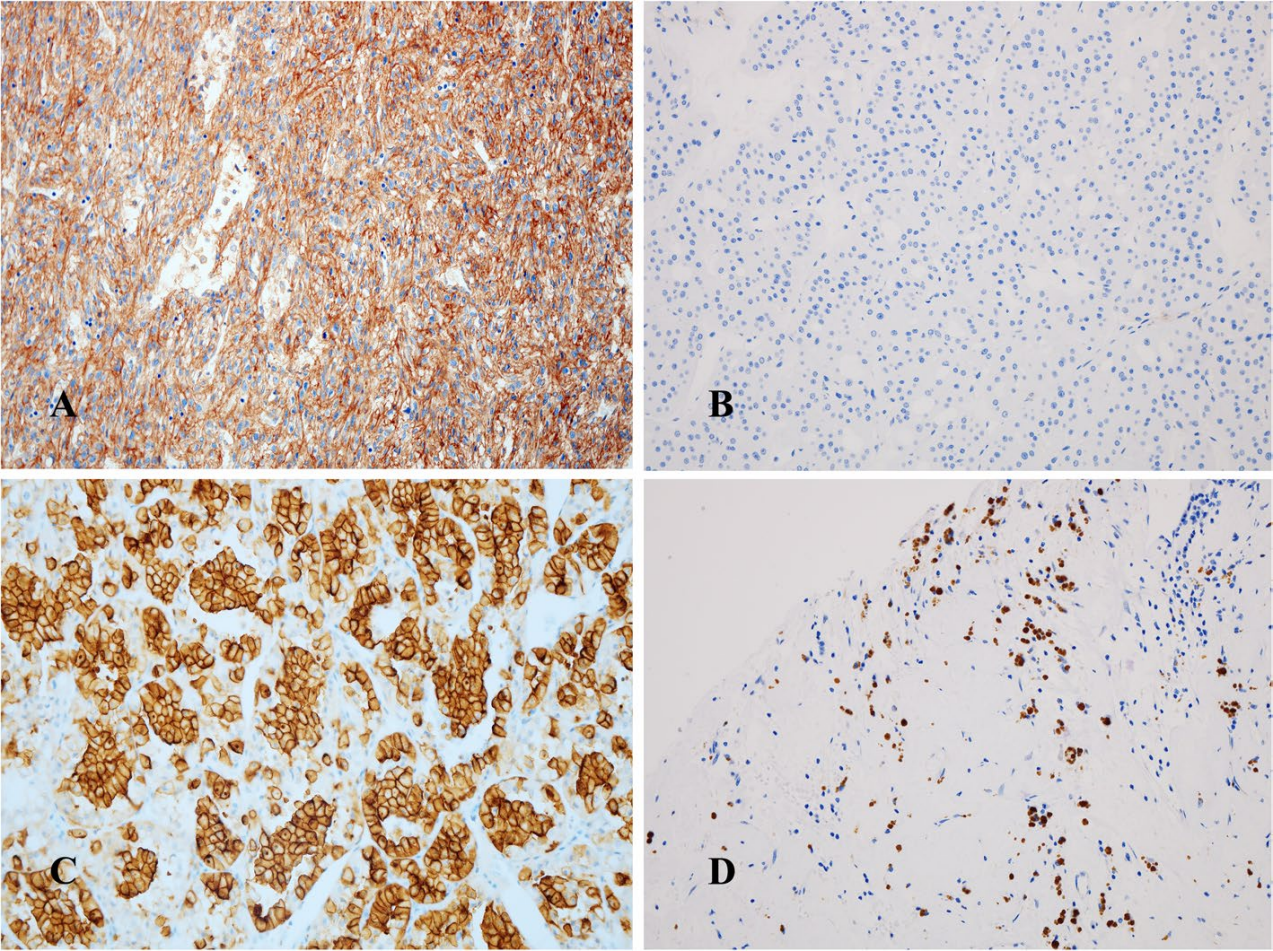
Immunohistochemical staining for B7-H3, PD-L1, and CTLA-4. B7-H3 positive staining (**A**); B7-H3 negative staining (**B**); PD-L1 positive staining (**C**); CTLA-4 positive staining (**D**)

Of the 244 patients, B7-H3 (CD276) expression was positive in 73 (29.9%) (Fig. 1A) but negative in 171 (70.1%) (Fig. 1B), and CTLA-4 expression was positive in 57 patients (23.4%) but negative in 187 patients (76.6%) (Fig. 1D). The expression of B7-H3 and CTLA-4 were correlated (χ^2^ test, *P* = 0.031, Table 2).

**Table 2:** Correlation between B7-H3 expression and morphologic and immunologic factors

Characteristic	Patient cohort of ccRCC		*P*-value
	B7-H3 Negative	B7-H3 Positive	
CTLA-4 expression			
Positive	33	24	0.031
Negative	138	49	
Metastasis			
Present	10	13	0.007
Absent	161	60	
Sarcomatoid pattern			
Present	5	11	0.001
Absent	166	62	
Necrosis			
Present	23	18	0.040
Absent	148	55	
PD-L1 expression			
Positive	17	24	**< 0.0001**
Negative	154	49	

### Morphological features and immunohistochemistry

**Fig. 2:**
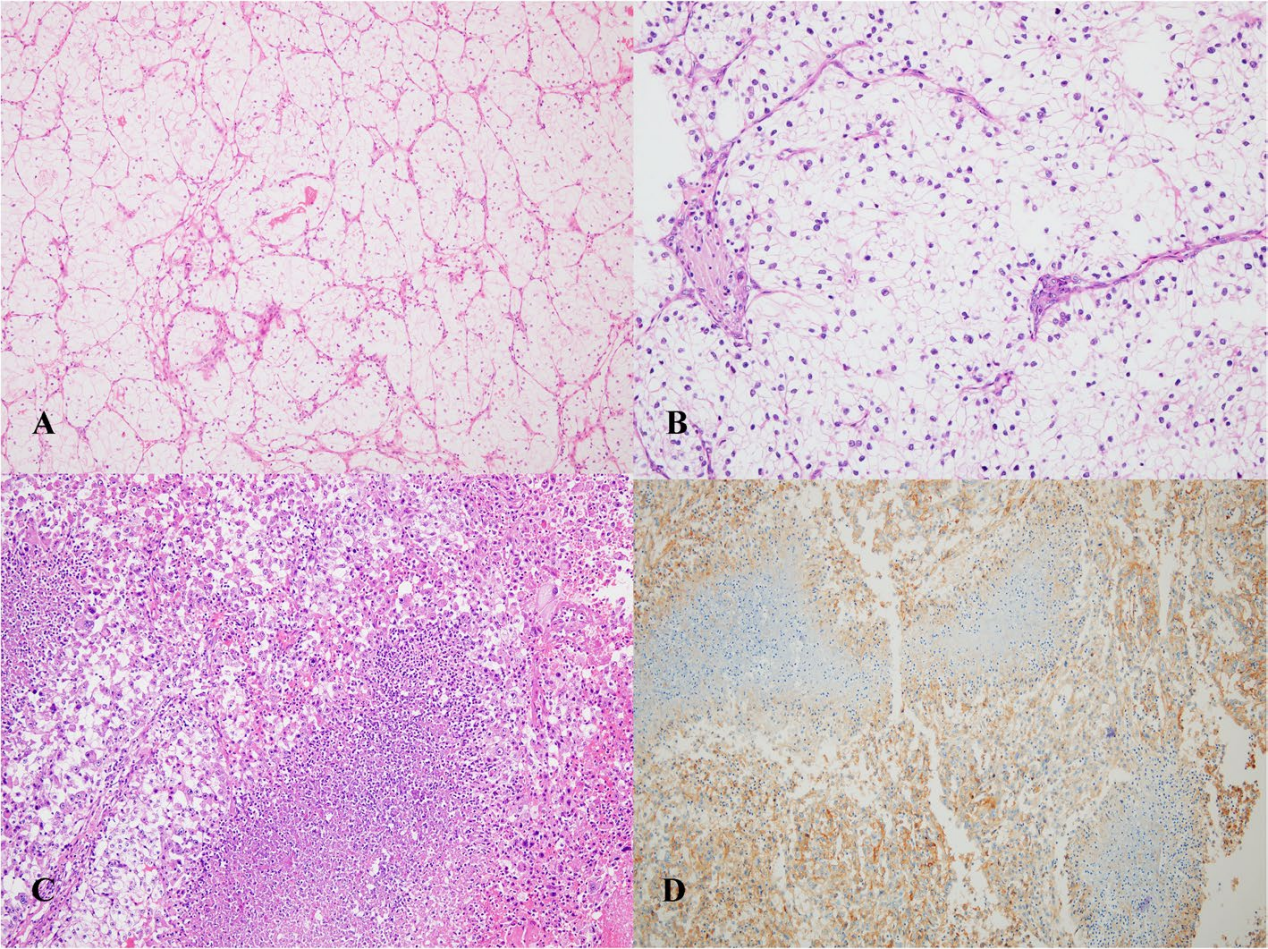
Renal cell carcinoma (RCC) cells stained with hematoxylin and eosin (H&E). Clear cell RCC (ccRCC) (**A**); immune cells with ccRCC (**B**); necrosis with a sarcomatoid pattern (**C**); .B7-H3 expression in sarcomatoid pattern (**D**)

B7-H3 expression was associated with a sarcomatoid pattern (*P* = 0.001, Table 2) (Fig. 2), whereas CTLA-4 expression was not (*P* = 0.769). Moreover, B7-H3 expression was associated with necrosis (*P* = 0.04), whereas CTLA-4 expression was not (*P* = 0.162).

### Comparison between B7-H3 and CTLA-4 expression and PD-L1 expression

χ^2^ tests revealed that relationships exist between PD-L1, B7-H3, and CTLA-4 expressions in the ccRCC cohort. B7-H3 expression was significantly associated with PD-L1 expression (*P* <  0.0001, Table 2) (Fig. 1C). However, CTLA-4 expression was not (*P* = 0.842).

### Relationship between B7-H3 and CTLA-4 expression and metastasis

B7-H3 positive staining was associated with metastasis (*P* = 0.007, Table 2). Similarly, a χ^2^ test was performed to determine the relationship between CTLA-4 and metastasis. No relationship was observed between the degree of CTLA-4 staining and metastasis (*P* = 0.118).

### Association between CTLA-4 and B7-H3 expression and PFS in ccRCC patients

**Fig. 3:**
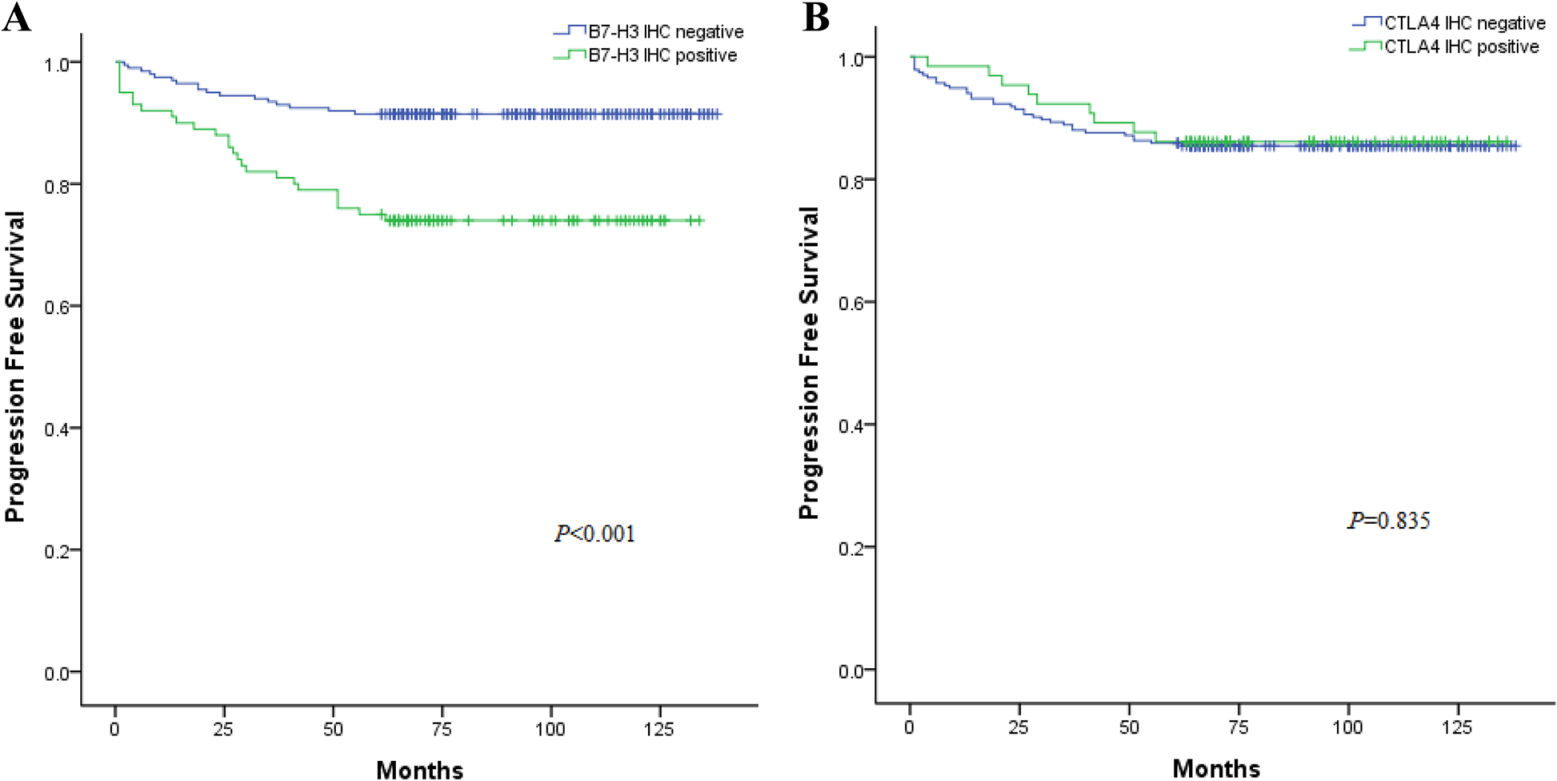
Progression-free survival (PFS) curves of RCC patients in the B7-H3 and CTLA-4-positive and -negative groups (Kaplan–Meier). The cases were divided into two groups showing positive or negative B7-H3 (**A**) and CTLA-4 (**B**) expression. The patients with positive B7-H3 expression showed poorer overall survival (*P* < 0.001) (**A**)

Kaplan–Meier analysis indicated that positive B7-H3 expression was associated with poor PFS (*P <* 0.0001) (Fig. 3A), whereas CTLA-4 expression was not (*P* = 0.457; Fig. 3B). Similarly, multivariate analysis revealed that B7-H3 was correlated with poor PFS (*P* = 0.031), whereas CTLA-4 was not (*P* = 0.173) (Table 3).

**Table 3:** Analysis of prognostic factors for survival

Variables	Univariate analysis		Multivariable analysis	
	HR (95% CI)	*P*-value	HR (95% CI)	*P*-value
Age > 50 years	2.07 (0.73, 5.85)	0.158	2.13 (0.74, 6.12)	0.161
Sex, female	0.83 (0.38, 1.82)	0.644	1.21 (0.53, 2.74)	0.650
ISUP Grade (3,4: High)	3.19(1.58, 6.46)	0.001	1.03 (0.43, 2.47)	0.944
Stage (III, IV: high)	8.08 (4.11, 15.89)	< 0.001	7.04 (3.05, 16.23)	< 0.001
High CTLA4 expression	0.73 (0.32, 1.67)	0.457	0.56 (0.24, 1.29)	0.173
High B7-H3 expression	3.03 (1.59, 5.79)	< 0.001	2.11 (1.07, 4.15)	0.031

## Discussion

ccRCC is recognized as an immunogenic tumor. Many studies have focused on immune-based approaches to ccRCC treatment. B7-H3 is a member of the B7 family of immune regulatory proteins that regulate T cell-mediated immune responses and is speculated to control tumor aggressiveness in various cancer types [18, 19]. Given its recent discovery, the regulatory mechanisms of B7-H3 are ill-understood. B7-H3 mRNA expression has been found in multiple human tissues and cell lines, such as prostate cancer, non-small-cell lung carcinoma, and RCC [20], and B7-H3 has been implicated as a potential inhibitor of T-cell activity [21]. B7-H3 has been noted to be expressed by dendritic cells and related with suppressive activity by contacting with CD4^+^CD25^+^ regulatory T cells [22]. B7-H3 ligand expression may be regulated by tumor microenvironment, and is supported by differential B7-H3 expression with different tumor types [23].

The expression of B7-H3 in tumor vascular endothelium and its clinical significance are gradually becoming important [24, 25]. B7-H3 could act as potent new cancer vessel-specific carrier to deliver antiangiogenic agents, and could help predict the clinical outcome of using different targeted agents in the treatment of ccRCC [26, 27].

The immunohistochemical staining of B7-H3 and CTLA-4 in 244 concurrent ccRCC cases treated with partial or total nephrectomy at Pusan National University (Yangsan, Korea) between 2011 and 2017 was conducted and analyzed. Our study has three main findings. First, one of the most important purposes of this study was to determine the correlation between the expression of the B7-H3 family members and ccRCC progression. Patients who were B7-H3 immunohistochemistry positive exhibited poor PFS (*P* < 0.001). In this study, a correlation was observed between the expression of the B7-H3 family members and necrosis, sarcomatoid pattern, and metastasis. Second, no correlation was observed between CTLA-4 expression and ccRCC progression. There were no correlations between CTLA-4 and necrosis, sarcomatoid pattern and metastasis. Third, B7-H3 expression was associated with PD-L1 expression, whereas CTLA-4 expression was not. Given that B7-H3 was correlated with poor prognosis in RCC, performing B7-H3 immunohistochemistry analysis will be useful in evaluating the prognosis of ccRCC patients.

This study is relevant as B7-H3 is a promising target for future immunotherapies. Immunotherapy using the B7-H3 pathway is effective when chemotherapy and radiotherapy are performed simultaneously. Additionally, the study findings will help elucidate the relationship between the B7-H3 pathway and cancer progression and ultimately facilitate the treatment of ccRCC.

CTLA-4 expression was not associated with ccRCC prognosis in this study, and the immunological processes mediated by CTLA-4 have not yet been clarified [28, 29]. Some studies have suggested that CTLA-4 expression positively correlates with cancer severity and prognosis [30]. We need to include more cases and further studies would help clarify the relationship between CTLA-4 and ccRCC.

## Conclusions

To the best of our knowledge, this study is the first to investigate the correlation between B7-H3 and PD-L1 expression and survival in ccRCC. B7-H3 expression is an independent prognostic factor for ccRCC. Furthermore, multiple immune cell inhibitory targets, such as B7-H3 and PD-L1, can be used for therapeutic tumor regression in a clinical setting.

## Data Availability

The dataset supporting the conclusions of this article is included within the article.

## Abbreviations

RCC: Renal cell carcinoma
ccRCC: Clear cell renal cell carcinoma
ISUP: International Society of Urological Pathology
CTLs: Cytotoxic T lymphocytes
TMAs: Tissue microarrays
PFS: Progression-free survival
