# Correction: B7-H3 expression is associated with high PD-L1 expression in clear cell renal cell carcinoma and predicts poor prognosis

**DOI:** 10.1186/s13000-023-01356-2

**Published:** 2023-05-16

**Authors:** Jung Hee Lee, Yong Jun Kim, Hyun Woo Ryu, Seung Won Shin, Eun Ji Kim, So Hyun Shin, Joon Young Park, So Young Kim, Chung Su Hwang, Joo‑Young Na, Dong Hoon Shin, Jee Yeon Kim, Hyun Jung Lee

**Affiliations:** 1 Department of Pathology, Pusan National University Yangsan Hospital, Pusan National University School of Medicine, Yangsan, Korea; 2 School of Medicine, Pusan National University, Yangsan, Korea; 3 The Research Institute for Convergence of Biomedical Science and Technology, Pusan National University Yangsan Hospital, Yangsan, Korea

**Correction: Diagnostic Pathology 18, 36 (2023)**

10.1186/s13000-023-01320-0

Following publication of the original article [1], the authors requested to update the affiliations to:

1. Department of Pathology, Pusan National University Yangsan Hospital, Pusan National University School of Medicine, Yangsan, Korea
2. School of Medicine, Pusan National University, Yangsan, Korea
3. The Research Institute for Convergence of Biomedical Science and Technology, Pusan National University Yangsan Hospital, Yangsan, Korea

The original article [1] has been updated.
